# Direct measurements of multi-photon induced nonlinear lattice dynamics in semiconductors via time-resolved x-ray scattering

**DOI:** 10.1038/srep39506

**Published:** 2016-12-22

**Authors:** G. Jackson Williams, Sooheyong Lee, Donald A. Walko, Michael A. Watson, Wonhuyk Jo, Dong Ryeol Lee, Eric C. Landahl

**Affiliations:** 1 Department of Physics, DePaul University, Chicago, Illinois 60614, USA; 2 Lawrence Livermore National Laboratory, Livermore, California 94550-9234, USA; 3 Frontier in Extreme Physics, Korea Research Institute of Standards and Science (KRISS), Daejeon 305-340, Republic of Korea; 4 Department of Nanoscience, University of Science and Technology (UST), Daejeon 305-350, Republic of Korea; 5 Advanced Photon Source, Argonne National Laboratory, Argonne, Illinois 60439, USA; 6 Department of Physics, Soong-Sil Univeristy, Seoul 06978, Republic of Korea

## Abstract

Nonlinear optical phenomena in semiconductors present several fundamental problems in modern optics that are of great importance for the development of optoelectronic devices. In particular, the details of photo-induced lattice dynamics at early time-scales prior to carrier recombination remain poorly understood. We demonstrate the first integrated measurements of both optical and structural, material-dependent quantities while also inferring the bulk impulsive strain profile by using high spatial-resolution time-resolved x-ray scattering (TRXS) on bulk crystalline gallium arsenide. Our findings reveal distinctive laser-fluence dependent crystal lattice responses, which are not described by previous TRXS experiments or models. The initial linear expansion of the crystal upon laser excitation stagnates at a laser fluence corresponding to the saturation of the free carrier density before resuming expansion in a third regime at higher fluences where two-photon absorption becomes dominant. Our interpretations of the lattice dynamics as nonlinear optical effects are confirmed by numerical simulations and by additional measurements in an n-type semiconductor that allows higher-order nonlinear optical processes to be directly observed as modulations of x-ray diffraction lineshapes.

The nonlinear optical properties of semiconductors remain a focus of growing scientific and technological interest because their intricate light absorption mechanisms have found a wide range of uses in optical communication and ultrafast lasers such as saturable absorbers^1^,^2^, all-optical switching^3^,^4^, fiber transport^5^ and control of photonic crystals^6^. Free charge carrier dynamics in nanocrystals and organic films have also drawn a great deal of attention due to their favorable optoelectronic properties for applications in high-speed transistors^7^,^8^, photovoltaics^9^,^10^,^11^, and light emitting diodes^12^. In such applications, kinetic energies of the relevant free carriers (~meV) are typically comparable to high frequency phonon (optical and acoustic) modes, and thus easily couple to the crystal lattice. These energy relaxation paths often lead to a rapid heating or deformation of the crystal structures through various scattering processes such as carrier-phonon interactions and Auger or trap recombinations. Under an extreme level of photoexcitation, they can even induce a nonlinear response of the lattice and potentially alter physical properties of the material such as carrier relaxation rates and diffusion velocities^13^,^14^. More recently, it has been reported that applying tensile strains on a single element semiconductor can bring a dramatic enhancement to its light emission capacity by altering the electronic band-structure of the material^15^. Therefore, continual developments of next generation devices will require precise understanding of a delicate interplay between the light induced electronic responses and mechanical/structural properties at various levels of photoexcitation. However, such effects of the free carriers on the crystal lattice are very difficult to quantify due to large differences between the electron and ion masses. While there have been extensive theoretical and optical experimental studies on these lattice dynamics, the interpretation of the results is complicated by both the difficulty of separating the free carrier from thermoelectric response as well as the determination of the depth-dependent strain profiles within the bulk crystal.

High-resolution time-resolved x-ray scattering provides a direct means to characterize such lattice dynamics by measuring ultrafast atomic movements on the surface as well as into the bulk of the material. During recent decades, with the advent of high brightness pulsed x-ray sources, it has become possible to probe atomic scale dynamics at the relevant length scales (micro- to femtometers) and time scales (sub-nanosecond)^16^,^17^,^18^,^19^,^20^,^21^. For instance, earlier work using TRXS on GaAs has demonstrated the feasibility of measuring the crystal lattice response upon heating at nanosecond regimes^22^ as well as dynamics of coherent phonon excitation and its propagation near the melting threshold^23^. More recently, an alternate effort has been made to deduce electron-lattice coupling mechanisms in GaAs by modeling data from x-ray pumped, transient optical reflectivity measurements at FEL facilities^24^. However, the levels of pumped fluences presented in these studies were very high (tens of mJ/cm^2^) and do not provide a full picture of how varying levels of excitation powers affect the evolution of crystal lattice responses. As a result of these experimental findings, it is now widely accepted that the deformation potential is usually the most important mechanism for carrier-acoustic-phonon interactions at low excitation intensities. Other studies have investigated the nature of “electronic melting” at extreme levels of laser-excitation, demonstrating the weakening of the interatomic forces due to a sudden deficit of valence electrons prior to lattice heating^25^,^26^,^27^. However, surprisingly little is known about the transient deformation of the solid structure at intermediate levels of electronic excitation during which several competing non-linear optical phenomena are taking place.

Here we quantitatively resolve and identify different stages of the structural and optical evolution of single crystal semiconductors under various levels of optical excitation, qualifying how distinct optical processes such as single-photon absorption (SPA), free-carrier saturation and two-photon absorption (TPA) each introduce unique effects on the crystal structure. For this TRXS study, we have selected a gallium arsenide (GaAs) direct band-gap polar semiconductor, which is widely used in the applications of transistors, optoelectronics^28^, and ultrafast terahertz spectroscopies^29^ because of its high-speed photo-carrier responses. Many studies have been performed to characterize and understand temporal evolution of GaAs properties upon intense optical laser excitation. For instance, several all-optical pump-probe experiments were carried out to investigate nature of high-speed melting^30^,^31^ and to characterise nonlinear optical absorption phenomena^32^. While numerous studies have been performed to characterize free carrier generation and relaxation dynamics in multi-photon absorption regimes, it is still primarily unknown how they alter lattice properties of the material. In this work, by directly probing high-speed transient movements of the lattice of the GaAs crystal below its plastic deformation limit, we find that the lattice response can be divided into three distinct regimes. Our findings show that the interatomic spacing of the crystal initially expands linearly with respect to the laser fluence; however, it eventually stagnates at a laser fluence corresponding to the saturation of the free carrier density. At even higher fluences, it resumes expansion in a third regime where two-photon absorption becomes significant. We also report observation of delayed lattice expansion in nanosecond time-scales, which is a signature of a slowing of carrier diffusion and recombination. These results demonstrate that using both optical and structural information derived from x-ray scattering, an accurate representation of the temporal and spatial dependence of the free carrier and lattice profile inside the crystal bulk can be measured. Such information and methodology hold fundamental interest in optical and condensed matter physics and may be useful to exploit nonlinear photo-physical properties of various materials.

## Time Resolved High Resolution X-ray Scattering

**Figure 1:**
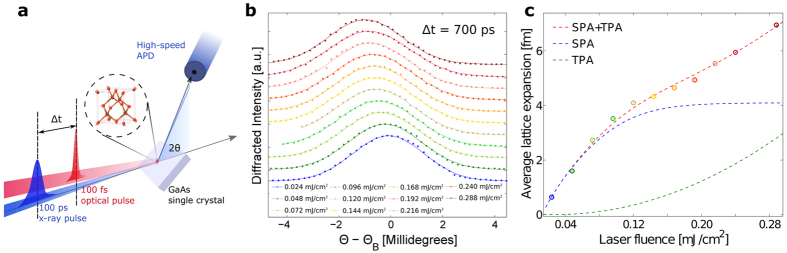
(**a**) Experimental schematics for TRXS measurement. (**b**) Bragg diffraction rocking curves at various laser fluences (vertically offset) at Δt = 700 ps are fitted to Gaussian distribution. (**c**) Mean lattice displacements, which are obtained by converting Θ − Θ_*B*_ into Δ*d* via Bragg’s law, along [004] reflection at Δt = 700 ps after the laser for varying laser fluences. The transient lattice response can be divided into three distinct regimes as the function of incident laser fluence, (i) linear expansion of the crystal, (ii) saturation of the linear response, and (ii) reoccurrence of a linear-like expansion at the highest fluences.

Our TRXS experiments were performed at Advanced Photon Source beam line 7ID-C^33^ to measure atomic scale changes in an undoped [001] surface oriented 300 *μ*m thick GaAs single crystal irradiated by a 100 fs laser pulse with a wavelength of 800 nm operating at a 5 kHz repetition rate. X-ray diffraction rocking curves were measured from the [004] symmetric lattice planes using a monochromatic 10 keV x-ray beam collimated to 50 *μ*m vertically and focused down to the equivalent size in the horizontal direction. Bragg-peak rocking curves with widths approaching the dynamical diffraction limit were measured where the centroids of each peak provide the mean lattice constant, integrated over the 1.57 *μ*m x-ray extinction depth, as a function of laser fluence and laser-to-x-ray time delay (Fig. 1a). Detailed peak shape analysis can be used to identify different lattice constants within the extinction depth. A half-wave plate followed by a polarized beam splitter was inserted in the laser beam path to control the fluence of the laser incident on the sample.

## TRXS Experimental Result and Interpretation

Ultrafast light absorption in direct bandgap semiconductors occurs through interband excitation of the valence electrons; the transient non-equilibrium states persist until the free electrons in the conduction band eventually recombine with holes. In GaAs, these photo-excited carriers exert positive electrostatic pressure on the lattice while also increasing the temperature through thermalization, primarily by carrier-carrier scattering over tens to hundreds of fs^34^. During that time, the sudden changes in the valence electron populations induce lattice displacements via acoustic deformation potential coupling^35^,^36^ where the presence of free carriers in the conduction band shifts the energy bandgap and exerts hydrostatic pressures whose magnitudes are proportional to the free carrier density. In TRXS measurements, structural deformation due to such high-speed lattice dynamics can be directly measured from changes of x-ray diffraction conditions. In Fig. 1b, Bragg diffraction peaks show the fluence-dependent lattice response to laser irradiation at maximum displacements (Δt = 700 ps). The overall shifts of the Bragg diffraction peak toward smaller angles indicates that the average lattice spacing of the crystal within the x-ray penetration depth expands further as the incident laser fluence increases. The resulting average atomic displacements can be extracted by converting the angular diffraction shifts, ΔΘ_*B*_, to changes in the lattice parameter, Δ*d*, using Bragg’s law, *λ* = 2(*d* ± Δ*d*(*t*)) sin (Θ_*B*_ 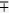 ΔΘ_*B*_(*t*)), where *λ* is the x-ray wavelength and *d*(*t*) and Θ_*B*_(*t*) are the temporally evolving crystal lattice spacing and Bragg diffraction angle, respectively. The diffraction angle is measured by extracting the centroid of a Gaussian fit to the rocking curve data, providing the aggregate lattice conditions over the finite x-ray extinction depth (see Fig. 1b).

**Figure 2:**
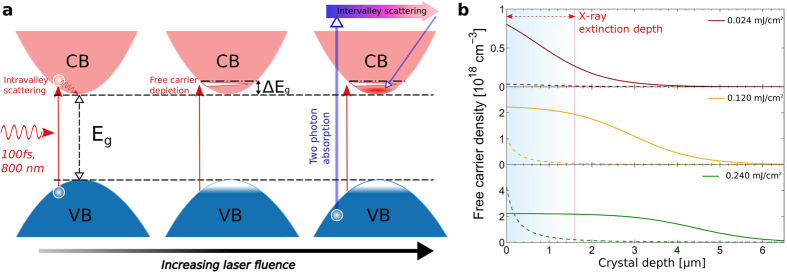
(**a**) Illustration of optically excited electronic energy bandgap. Free carriers fill the available electron density of states near the conduction band edge. As the laser fluence increases, the free carrier concentration exceeds the density of states in the conduction band edge, effectively increasing the energy bandgap by ΔE_*g*_. Finally, under extreme level of optical excitation, a simultaneous absorption of two photons excites a valence band electron to a high energy state such as *L* valley, which eventually relaxes down to the Γ valley via intervalley scattering. In the atomic-scale, as the number of incident photons increases, the likelihood of TPA also increases despite its small photon-electron cross-section as compared to SPA events. As illustrated in Fig. 2a (right), when two photons simultaneously promote a valence band electron to a low-mobility satellite valley (e.g., X-valley), the electron immediately decays to the Γ-valley^50^. This intermediate electron-transition process occurs on time scales that are much faster than the time-resolution of the x-ray probe available in this experiment. However, since the carrier population is linearly related to the volumetric lattice expansion via the deformation potential coupling coefficient, we can directly estimate the carrier density as a function of laser fluence from the measured lattice-displacement at Δt = 700 ps, where the maximal displacements occur due to the presence of impulsively generated strain within the x-ray probe depth. (**b**) Calculated free carrier distribution for three different laser fluences of 0.024, 0.120 and 0.240 mJ/cm^2^. Each profile displays unique traits of the SPA (solid lines) including saturation as well as the TPA process (dashed lines).

A Gaussian fit to these rocking curves show regimes of (1) a linear increase, (2) a fall-off in the slope (strain saturation), and (3) the recurrence of a linear-like response with respect to the incident laser fluence (Fig. 1c). We interpret the first linear-response regime as the generation of free carriers below the single photon absorption saturation regime (Fig. 2a (left)). For instance, the peak of the longitudinal strain value 1.14 × 10^−5^ measured at the laser fluence of 0.048 mJ/cm^2^ yields the deformation potential (i.e. difference in electron and holes) coupling coefficient of −2.02 eV, which is comparable to DFT calculations of −2.44 eV for GaAs^37^. However the appearance of the strain-saturation at moderately higher laser power indicates that increasing the laser fluence no longer efficiently generates free carriers inside the material.

In the “saturation” regime, when the pumping laser fluence increases, the excited carrier density becomes comparable to the available electronic density of states, of which energy and bandwidth are determined by the spectral properties of the incident laser pulses as illustrated in Fig. 2a (middle). Since the fermions cannot occupy the same energy state, the lack of promotable states eventually leads to a bleaching of the front surface of the crystal. Upon photoexcition, the population conversion threshold at room temperature for the available states in the conduction band is approximately 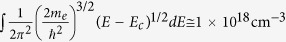, where *m*_*e*_ and *E*_*c*_ are the effective mass of carriers and the conduction band edge of the material. Details of this phenomenon can be described via the dynamic Burstein-Moss effect^38^, in which the apparent bandgap of a semiconductor is effectively increased as the absorption edge is pushed to higher energy,


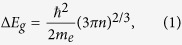


where Δ*E*_*g*_ represents the effective increase of the energy bandwidth and *n* is the free carrier density. The bandgap of GaAs (1.424 eV) is only 0.13 eV less than the photon energy. Since the density of states near the conduction band is relatively low, the dynamic Burstein-Moss shift leads to drastic bleaching even at low incident pulse energies. As a result, GaAs exhibits a significant increase in transparency (i.e., SPA coefficient), and the carrier density can saturate over multiple absorption lengths. Here it is very interesting to note that redistribution of the electrons following the dynamic Burstein-Moss effect occurs within 1.3~1.7 ps in GaAs^38^, which is much faster than the time-resolution of our TRXS method. Since the crystal lattice only relaxes on the time scale of recombination, TRXS measurement is sensitive to these optically-driven processes, which is made possible due to the femtometer (1 × 10^−15^ meter) spatial resolution provided by high-resolution x-ray diffraction at synchrotrons. At higher fluences, >0.1 mJ/cm^2^, the probability of two-photons being absorbed by an ion increases, which generates free carriers with greater kinetic energies (see Fig. 2b (right)).

To approximate such nonlinear free carrier dynamics, we employed a saturable absorber model, where the absorption depends on the incident photon intensity^39^ and a total carrier density due to SPA is given as


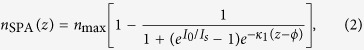


where *I*_0_, *I*_*s*_, *κ*_1_, and *ϕ* are incident photon intensity, saturation intensity, linear absorption coefficient, and a fitting parameter that represents the depth into the crystal of carrier depletion region, respectively. While Eq. 2 predicts carrier profiles inside the crystal up to the free carrier saturation regime, it is still insufficient to explain re-emergence of lattice expansion beyond the critical value of 0.2 mJ/cm^2^. We correlate the increased strain above the saturation limit to the additional free-carrier population that is generated via the TPA process. In general, the instantaneous light intensity *I*(*z*) passing through a bulk material is described by single and multi-photon absorption processes, in which the laser intensity dependent absorption mechanism most likely occurs via TPA^40^


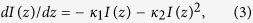


where *κ*_1_ and *κ*_2_ are single-and two-photon absorption coefficient respectively. TPA is expressed by carrier density predicted as 
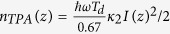
 where *ω* is the angular frequency of the pump pulse and *T*_*d*_ is the pulse duration^39^. Its role is most important at high intensities that are still below the damage threshold.

In Fig. 2b, we compare the instantaneous free carrier profiles at Δt = 0 that are each generated from the SPA and TPA processes near the crystal surface upon laser excitation. At a relatively low laser fluence of 0.024 mJ/cm^2^, the model based on Eq. 2 shows a near exponential decay of the free carrier population along the crystal depth with a marginal TPA contribution. At an intermediate laser fluence of 0.120 mJ/cm^2^, the onset of the carrier saturation can be expected, in which the free carrier depleted depth (plateau region from the surface) is nearly uniform over the length of the x-ray extinction depth of 1.57 *μ*m. Finally as the laser fluence increases to 0.24 mJ/cm^2^, the quadratic increase of the TPA effect begins to overtake the SPA counterpart, especially near the surface. In this “TPA dominating region,” the carrier depleted region extends even further, up to 3~4 *μ*m into the crystal. The blue and green dashed lines in Fig. 1c each show numerical fits of the SPA and TPA contributions to the average lattice expansions at Δt = 700 ps. Coincidently, we note that the average lattice expansion rate (slope of Fig. 1c) is doubled between the low fluence (SPA dominated region) and high fluence (TPA dominated). This observation implies that significantly more incident photons in the TPA regime are required for each unit increase in electrostatic pressure and volumetric lattice expansion. When the effects of each absorption processes are superimposed, we are able to reproduce the nonlinear lattice expansion behavior, which is proportional to the integrated number of free carriers within the x-ray extinction depth based on the laser fluences.

## Numerical Modeling

**Figure 3:**
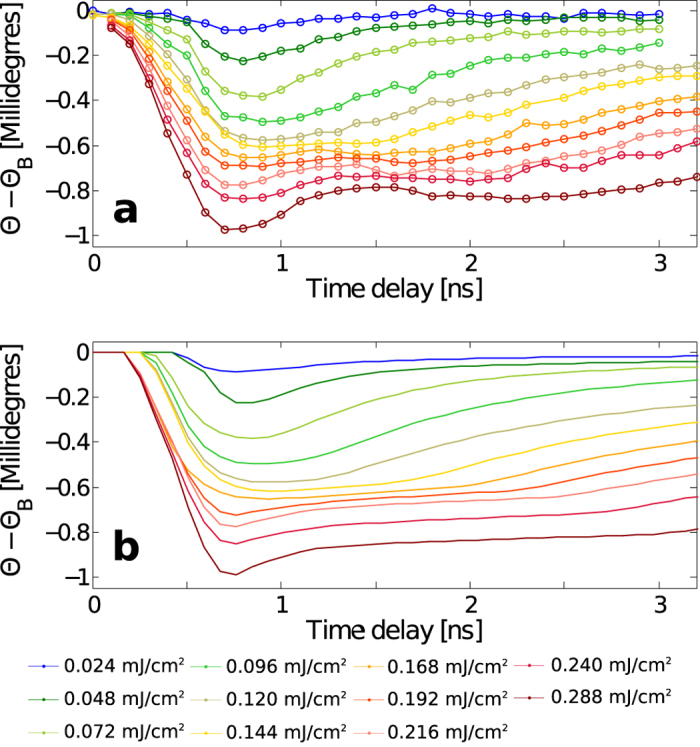
(**a**) The angular shifts Θ − Θ_*B*_ are calculated by taking the differences in the (0 0 4) diffraction peak centroid before and after the laser strikes the sample as a function of x-ray to laser delays for varying laser fluences. (**b**) Simulated angular shifts Θ − Θ_*B*_ for the (0 0 4) diffraction peak after effects of carrier saturation and TPA are incorporated, showing consistent results with the experimental data above.

Direct comparison between x-ray scattering data and the simulation enables an understanding of nonlinear lattice responses as well as extracting important parameters such as *κ*_2_ and the extent of the carrier depletion regions. We note that there is a considerable difference between the penetration depths of the optical pump and x-ray probe pulses. In order to account for the penetration depth-mismatch, our numerical modelling utilizes dynamical theory of x-rays^41^ that simulates x-ray diffraction peaks for depth-dependent strain profiles as a function of the x-ray penetration depth (See TRXS Simulation in METHODS). Measured aggregate lattice displacements from equilibrium are shown in Fig. 3a for varying incident laser fluences that exhibit three qualitatively distinct patterns. For low fluences (up to 0.072 mJ/cm^2^), the lattice impulsively expands and begins to equilibrate immediately. Here, free carriers quickly recombine and diffuse away from the x-ray penetration depth, leaving only a small residual lattice expansion due to a temperature increase. Moderate laser fluences of 0.096–0.192 mJ/cm^2^ saturate the free carrier density at the front surface, which prolongs the time (0.1–1 ns) that carriers are present at the surface, leaving recombination as the primary means by which the electrostatic pressure is reduced and thereby delaying the lattice recovery by up to a nanosecond. This observation indicates that the strain profile can no longer be described by a simple exponential decay. Instead the laser absorption profile has a spatial extent several factors greater than the x-ray penetration depth of 1.57 *μ*m, which corresponds to the width of the carrier depleted region previously shown in Fig. 2b. The altered initial carrier profile results in a strain profile with an impulsive component whose wavelength is larger than the extinction depth, thereby changing the lattice response shape characteristic compared to the low fluence cases. Subsequently, the lattice recovery is then dominated by relatively slow electron and thermal diffusion. At the highest fluences presented here (0.216–0.288 mJ/cm^2^), impulsive recovery and immediate equilibration reappears, although superimposed over the stagnated expansion observed for the moderate fluences. In this regime, TPA becomes the dominant source of free carriers.

For a more quantitative interpretation, we incorporated these modified light absorption profiles into our TRXS simulation to reproduce some of the important features that are measured in our experiments. Details of the simulations are elaborated in the Methods section as well as in our previous studies^42^. Generation of free carriers by SPA and TPA shifts the energy bandgap and exerts sudden hydrostatic pressure on the crystal lattice via the deformation potential scattering coefficient *α*_*p*_^35^,^36^, which is proportional to the carrier density *n*_*e*_(*z, t*). Carrier diffusion, radiative and Auger recombination, and thermal expansion also play significant roles in the evolution and transportation of strain into the crystal bulk and are incorporated into the strain model^43^.

Figure 3b shows our TRXS simulation result after incorporating the modified initial strain profile (Eqs 2 and 3) to account for both the SPA saturation and TPA contributions. The calculation was matched to the data using the single free parameter *ϕ* in Eq. 2, and yielded a value for *κ*_2_ = (3.7 ± 0.6) × 10^−8^ cm/W that is consistent with previously reported values^39^,^44^. Slight deviations between the data and the simulation are observed near Δ*t* = 2 ns for the 0.288 mJ/cm^−2^ data set, in which the experimental data shows additionally increased angular shifts, a sign of a delayed lattice expansion. The oscillatory-like behavior in Fig. 3a shows an emergence of a new systematic signature of the lattice response at higher fluence regimes. However we currently lack a theory or model to explain this phenomenon, which remains a task for future studies. This observation raises questions about the nature of delayed interactions between free carriers and lattice modes and the relevant thermophysical properties such as Auguer recombination, carrier and thermal carrier diffusion speed at extremely high excitation fluences. This discrepancy might be attributed, for instance, to the effect of enhanced Auger recombination and slowing of carrier diffusion speed at extremely high carrier density. These questions could be addressed in the future by probing details of carrier generation and carrier-to-lattice coupling processes at X-ray free electron sources^45^,^46^, which are capable of producing femtosecond x-ray pulses. Such results would motivate the development of a more elaborate carrier recombination and diffusion model.

## Comparison with N-type Gallium Arsenide

**Figure 4:**
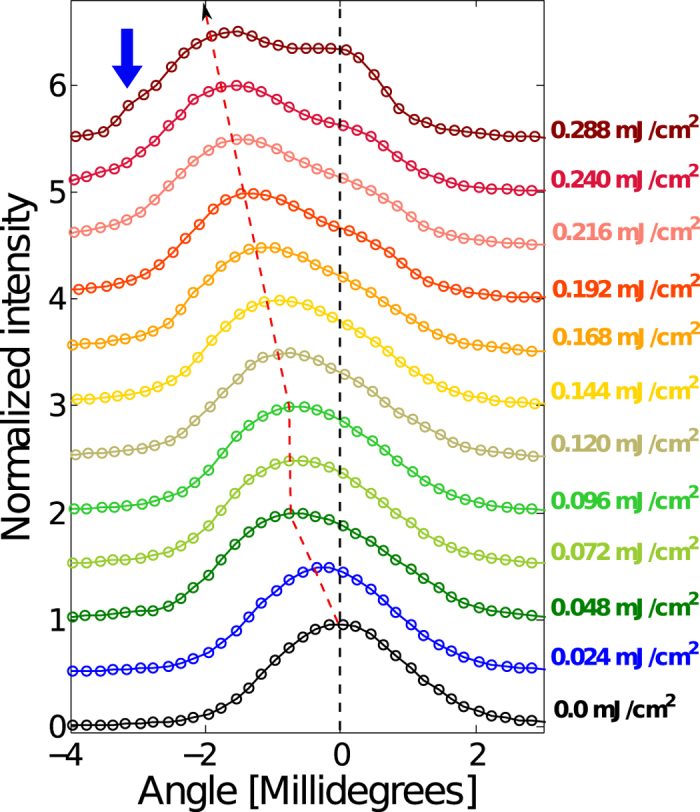
Bragg diffraction curves at various laser fluences (vertically offset) at Δt = 700 ps for the n-doped GaAs sample. Black dashed line indicates the Bragg diffraction condition for an unperturbed crystal. Red dashed line shows centroid of the rocking curve up to laser fluence of 0.048 mJ/cm^2^, and then subsequently the centroids of the de-convolved peaks that come from the laser excited part of the crystal.

We also performed TRXS measurements on an n-type GaAs sample, which was doped with Tellurium atoms at an extrinsic carrier concentration of ~1 × 10^18^ cm^−3^. In an n-type semiconductor, there exist intermediate electron energy levels near the top of the electronic bandgap, such that the electrons can be readily promoted from the energy level of the dopant into the conduction band at room temperature. These mobile electrons (carriers) now occupy large amount of available states in the conduction band. Consequently, we should expect that the “lattice saturation behavior” should occur at lower laser fluences due to a lack of the available density of states. Figure 4 shows the displaced Bragg diffraction peaks at Δt = 700 ps for the n-type sample. Initially, the positions of the Bragg peaks shift toward smaller angles (i.e. lattice expansion) linearly with the laser fluence as indicated by the red dashed line. Subsequently, the shifts of the peak position clearly stagnates between 0.048 and 0.072 mJ mJ/cm^2^ that is considerably lower than the case of the un-doped GaAs sample, which was found at ~0.12 mJ/cm^2^. This observation supports our initial interpretation of the laser induced strain saturation, which is apparently driven by the electronic responses of the material.

Above the saturation fluence, we observe a very interesting feature in the shapes of the diffraction peaks. As the incident laser power is increased further, the lattice expansion behavior resumes as it was shown in the un-doped data set. However, unlike in the case of the un-doped GaAs, the appearance of the diffraction peak progressively becomes more asymmetric, and eventually above 0.168 mJ/cm^2^ resolves into two peaks whose distinct amplitude and separation depend on the incident laser power. The emergence of these two diffraction peaks implies that there are two distinct layers that carry different lattice parameters along the crystal depth. One of the de-convolved peaks is positioned at the Bragg condition of the GaAs crystal near equilibrium (indicated by a black dashed line) while the other continuously shifts toward smaller angles as marked by the red dashed line. According to our TRXS modeling, such splitting of the peak is possible when the extent of the near-surface expanded layer is limited to ~100 nm from the surface, which is considerably shorter than the x-ray extinction depth. This is also consistent with TPA, which is mostly confined to the regions of highest fluence near the surface. The early onset of SPA saturation in the n-doped sample results in a narrower diffraction peak, allowing the onset of TPA to be more readily observed modifications of the peak shape. Consequently, we are visualizing the nonlinear progression of crystal lattice behaviors, during which its optical properties are changed from initially opaque to transparent to even more opaque. A subtle but important feature can be found in the diffraction peak profile at a laser fluence of 0.288 mJ/cm^2^ as indicated by blue-arrow in Fig. 4. Under the highest level of optical excitation performed in this study, we not only measure the distinct separation of the two peaks, but also an emergence of an additional third peak at (Θ − Θ_*B*_) ~3 millidegrees (marked by a blue arrow (B)). Such observation implies an additional layer that is even more opaque, which may be attributed to higher order optical absorption processes. We plan to re-visit details of this phenomena in future. Coincidently, it is very interesting to note that similar fluence-dependent lattice behavior has been previously reported by Basak *et al*.^47^, in which amplitude, frequency and dephasing behaviors of coherent phonons inside n-doped GaAs were studied as a function of the pump laser fluence. In the study, a saturation threshold for excitation of coherent LO phonons was found at about 3 × 10^19^ cm^−3^ (equivalent to a laser fluence of 0.48 mJ/cm^2^), which is slightly above our saturation fluence. Also closely related to this work, Kadlec *et al*.^39^ used THz spectroscopy to observe the interplay between SPA, TPA, and saturable absorption on the carrier density depth following intense laser excitation of GaAs just above the bandgap. Due to limited spatial resolution of a few microns, they required fluences of over 1 mJ/cm^2^ to observe the onset of TPA, compared to our observations that are sensitive at the scale of the optical penetration depth (~100 nm) where TPA was observed at fluences as low as 0.2 mJ/cm^2^.

## Conclusion

In summary, we performed TRXS measurements with sub-atomic length scale precision on the combined effects of strain saturation and complementary TPA effects in a solid by using synchrotron x-rays. We find that the deformation potential scattering is the fundamental and dominant reason for the impulsive responses of the lattice to the laser during the presence of a dense population of free carriers in GaAs. We also demonstrate multiple distinct crystal lattice behaviours can be manifested due to SPA, saturable absorption, and TPA. The quantitative agreement existing between the (0 0 4) reflection data sets and the simulation supports this interpretation. At even higher laser fluences, we would expect the significant amount of transverse kinetic energy present to enhance both nonlinear and anisotropic properties such as the deformation potential tensor and phonon softening^34^. We may also gain new physical insights from studying systems with very different electronic behaviors such as indirect bandgap semiconductors or semiconductors with even faster recombination times such as InSb. Finally, the extension of these methods to sub-picosecond x-ray capabilities at XFELs^45^,^46^ should allow the study of the intervalley transition process^48^, which takes place in time scales that are much faster than what can be resolved with x-rays from storage ring based sources.

## Methods

### Time-resolved x-ray scattering

Synchrotron TRXS was performed at Beamline 7ID of the Advanced Photon Source^33^ where a water-cooled double-crystal diamond (1 1 1) monochromator combined with horizontal-plane focusing and vertical slits provided a collimated, 50-micron square 10-keV x-ray beam on the semiconductor sample. Before and after the diffraction measurements, knife-edge scans of both laser and x-ray beams at the sample position verified that the laser uniformly overfilled the x-ray spot with a 5-mm FWHM smooth spatial profile. With the use of four-circle diffraction geometry, the sample was oriented in the x-ray beam such that the (0 0 4) reflection was measured near its intrinsic angular resolution. X-ray intensity was measured both before the sample from an upstream scattering polyimide foil for normalization as well as 1 m after the sample on the diffractometer arm, which used collimating slits to reduce background scatter. Absorbing filters were also placed on the detector arm to reduce the x-ray intensity so that an extended dead-time model^49^ could be used for dead-time correction and error estimation. Both x-ray detectors were single photon counting avalanche photodiodes capable of timing discrimination between successive x-ray pulses (separated by 153 ns). The amplified laser pulses were synchronized to the x-ray beam such that arbitrary laser–x-ray delay times, *t*, could be adjusted electronically with <1 ps precision and directed onto the sample surface colinearly with the x-ray beam.

### TRXS Simulation

We employ a one-dimensional carrier-driven strain model^43^ for laser-induced strain generation and propagation inside a GaAs bulk crystal. In our model, two assumptions are made: (i) the energy relaxation from femtosecond irradiation to creating free-carrier population takes place instantaneously, and (ii) the electron-hole pairs relay their energy immediately to the lattice. These are reasonable assumptions for the simulation because such processes take place within a few picoseconds, which is well beyond the time-resolution available at storage-ring based x-ray synchrotrons. We assume that the energy difference between the laser photon energy and the electronic-energy bandgap of the material (i.e., *E*_*p*_** − ***E*_*g*_) is transferred to the lattice heating. Here the initial laser induced free-carrier *n*(*z, t* = 0) near the surface is given by the carrier distribution profiles discussed in the TRXS experimental result section in the manuscript. The initial lattice temperature profile *T*(*z, t* = 0) is given by 
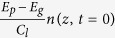
 where *C*_*l*_ is the crystalline heat capacity. Subsequent transient removal of excess carriers and diffusion of carriers and heat are characterised as follows:


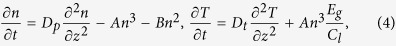


where *A, B, D*_*p*_, and *D*_*t*_ are Auger recombination rate, radiative recombination rate, ambipolar diffusion, and thermal diffusion coefficients respectively. Finally the strain contributions from the electronic pressure (generated via deformation potential) and temperature are given as via the volume deformation potential, *α*_*p*_ and thermal expansion *α*_*t*_ coefficients as follows:


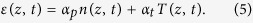


Rapid expansion of the lattice near the surface launches two counter propagating compression waves along the surface normal direction, resulting in coexistence of relatively slow-decaying electronic strain and two-mobile strains within a few nanosecond time-scale. To calculate the shift and shape evolution of the x-ray diffraction patterns for each laser fluence, we follow the numerical formulation initially derived by Wie *et al*.^41^, in which x-ray diffraction curves with depth-dependent strains fields are calculated by solving the Takagi-Taupin equation. In our simulation, the size of the incremental layer spacing is kept sufficiently thin such that the maximal strain change within a single lamina does not exceed 3 × 10^−8^ meters per time step. Tables 1 and 2 shows the list of parameters for the semiconductor material and x-ray scattering that are used for the simulation.

## Additional Information

**How to cite this article**: Williams, G. J. *et al*. Direct measurements of multi-photon induced nonlinear lattice dynamics in semiconductors via time-resolved x-ray scattering. *Sci. Rep.* **6**, 39506; doi: 10.1038/srep39506 (2016).

**Publisher's note:** Springer Nature remains neutral with regard to jurisdictional claims in published maps and institutional affiliations.

## Figures and Tables

**Table 1:** GaAs material parameters.

Lattice parameter	0.565325 nm
Energy bandgap	1.42 eV
Auger recombination rate	4 × 10^−38^ cm^6^/ns
Radiative recombination rate	1.7 × 10^−19^ cm^3^/ns
Deformation potential coefficient	5 × 10^−24^ cm^3^
Ambipolar diffusion coefficient	1 *μm*^2^/ns
Thermal diffusivity	0.03 *μm*^2^/ns
Crystalline heat capacity	1.13 × 10^19^ eV/cm^3^ K
Longitudinal sound speed	4.73 cm/s
Transverse sound speed	3.35 cm/s

**Table 2:** X-ray scattering parameters.

Reflection (h k l)	(0 0 4)
Photon energy (keV)	10.0
Absorption factor [1/cm]	348.5
Absorption depth [*μ*m]	24.0
Extinction depth [*μ*m]	1.57
F_*o*_	247.1 + 7.2i
F_*hkl*_	150.4 + 7.0i
